# 24-hour average PM2.5 concentration caused by aircraft in Chinese airports from Jan. 2006 to Dec. 2023

**DOI:** 10.1038/s41597-024-03110-9

**Published:** 2024-03-09

**Authors:** Qiang Cui, Zike Jia, Yujie Liu, Yu Wang, Ye Li

**Affiliations:** 1 School of Economics and Management, Southeast University, Nanjing, China; 2 School of Economics and Management, Civil Aviation Flight University of China, Guanghan, China; 3 School of Business Administration, Nanjing University of Finance and Economics, Nanjing, China

**Keywords:** Environmental impact, Environmental impact, Sustainability

## Abstract

Since 2006, the rapid development of China’s aviation industry has been accompanied by a significant increase in one of its emissions, namely, PM2.5, which poses a substantial threat to human health. However, little data is describing the PM2.5 concentration caused by aircraft activities. This study addresses this gap by initially computing the monthly PM2.5 emissions of the landing-take-off (LTO) stage from Jan. 2006 to Dec. 2023 for 175 Chinese airports, employing the modified BFFM2-FOA-FPM method. Subsequently, the study uses the Gaussian diffusion model to measure the 24-hour average PM2.5 concentration resulting from flight activities at each airport. This study mainly draws the following conclusions: Between 2006 and 2023, the highest recorded PM2.5 concentration data at all airports was observed in 2018, reaching 5.7985 micrograms per cubic meter, while the lowest point was recorded in 2022, at 2.0574 micrograms per cubic meter. Moreover, airports with higher emissions are predominantly located in densely populated and economically vibrant regions such as Beijing, Shanghai, Guangzhou, Chengdu, and Shenzhen.

## Background & Summary

As is universally acknowledged, air pollution has become one of the leading causes of death throughout the world, even surpassing tobacco smoking, HIV/AIDS, parasitic, vector or other-borne infectious diseases, and all kinds of violence^1^. PM2.5, as an essential component of air quality detection, also has a tremendous negative impact on human health and life expectancy^2,3^. It is estimated that PM2.5 causes millions of deaths annually and poses a significant threat to public health^4–8^. This particulate matter can infiltrate deep into the bronchioles and alveoli of the lungs, thereby precipitating respiratory ailments such as asthma, chronic obstructive pulmonary disease (COPD), and bronchitis^9^. Furthermore, PM2.5 has the capacity to enter the bloodstream, leading to the onset of cardiovascular conditions including heart disease, myocardial infarction, and stroke^10^. Additionally, prolonged exposure to elevated levels of PM2.5 has been associated with exacerbations of asthma, cardiac arrhythmias, acute myocardial infarction, and instances of sudden death^11^. It was noted that there were 185.7 million premature deaths caused by global anthropogenic PM2.5 pollution, with an average of 2.9 million each year. Specifically, higher-income groups tended to have higher death rates^12^. Moreover, seniors, females, and those with education attainment of primary school or below were found to have generally higher effect estimates of total mortality than their counterparts^13^. Therefore, from the perspective of health protection, it is vital to conduct in-depth research on PM2.5.

With the development of the aviation industry, aviation emissions have increasingly become an essential factor affecting air quality. Consequently, it is of increasing magnitude to quantify PM2.5 in aviation emissions. Many scholars have done a lot of research on PM2.5 emissions from aviation, which was demonstrated that within a single airport annually^14^, noting that the total emissions of PM from Xinzheng International Airport (CGO) from aircraft and ground support equipment (GSE) were 36.2 tons and the main engines of the aircraft accounted for 74.3% of the total emissions. Others have measured the amount and proportion of PM2.5 in aviation emissions across the country^15^. The proportion of PM2.5 in aviation emissions is about 0.67%, along with a growing trend in quantity and a declining inclination in the growth rate^15–19^.

Dozens of models have been made to measure PM2.5 concentration, and scholars have adopted different methods for different research topics. Currently, there are three categories of models to quantify the concentration of PM2.5. First, the U.S. Environment Protection Agency (EPA) ‘s first generation of air quality models, which are divided into box model, Gaussian diffusion model and Lagrange trajectory model. Specifically, the Gaussian diffusion model^20,21^ includes Industrial Source Complex Short-Term (ISCST3)^22,23^, the American Meteorological Society (AMS) and EPA Regulatory Model (AERMOD)^24,25^, the Atmospheric Dispersion Modelling System (ADMS)^26^. Second, scholars have applied the comprehensive regional scale models such as the Nested Air-quality Prediction Modeling System (NAQPMS)^27–30^, the Comprehensive Air Quality Model with Extensions (CAMX)^31,32^, the Weather Research and Forecasting-Chemistry (WRF-CHEM)^33–35^ and the Community Multiscale Air Quality (CMAQ)^36–40^ for measurement in more complicated situation, which provide a “one atmosphere” perspective to demonstrate the various atmospheric physical processes and chemical reactions among various pollutants and gas-solid two-phase conversion processes. Third, with the deepening of globalization, more and more scholars take global pollution problem into consideration, which contributes the application of global large-scale models, such as the Model for Ozone and Related chemical Tracers (MOZART)^41–43^ and the Goddard Earth Observing System-Chemistry (GEOS-CHEM)^44–49^. Both are mainly used to simulate the long-distance transportation and chemical reaction process of global scale air pollutants. Among these models, the Gaussian diffusion model stands out for its ease of operation, faster calculation speed, lower data requirements, and high accuracy in simulating long-term concentrations. Therefore, it is extensively employed in simulating conventional pollutants.

**Fig. 1:**
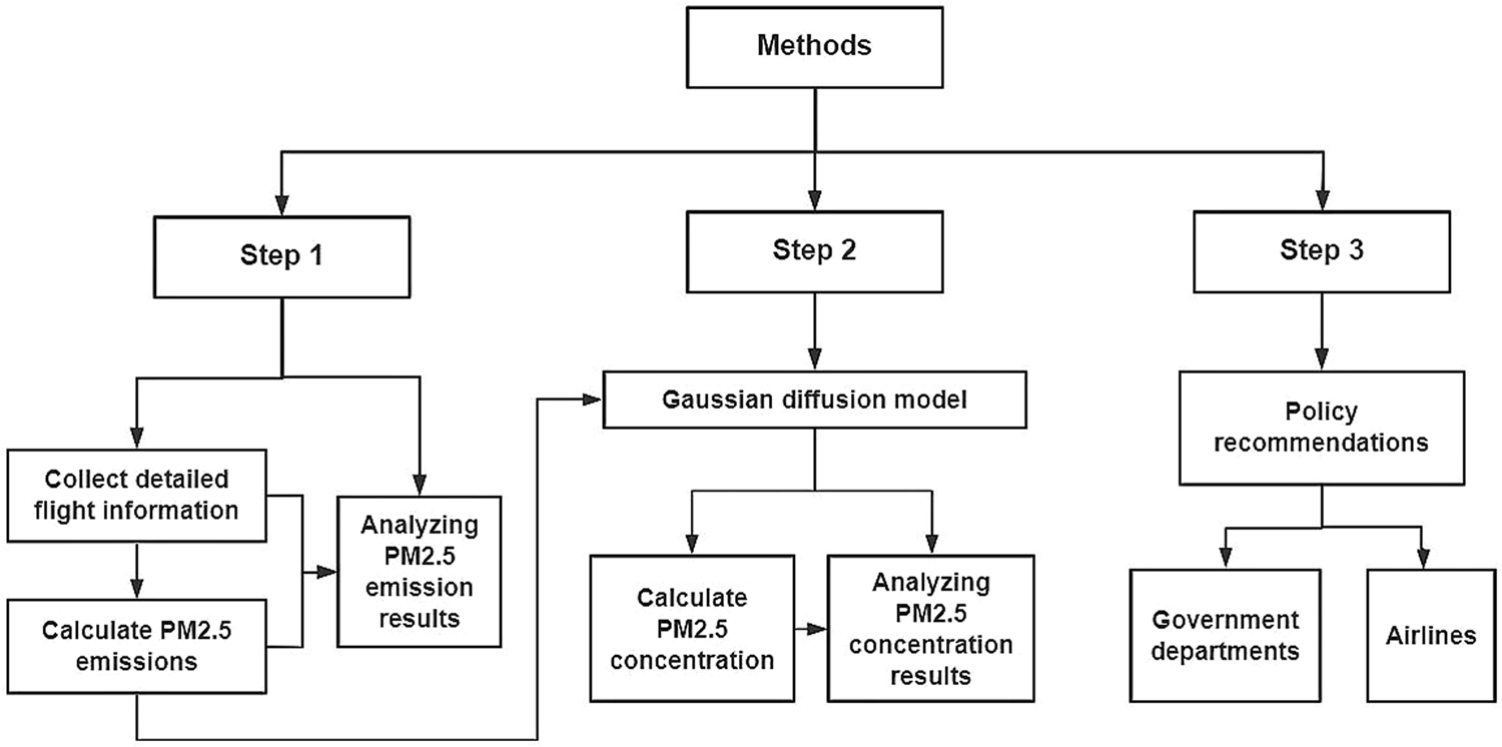
The research framework diagram of the methods.

In summary, PM2.5 is a significant component of aviation emissions, which may cause various diseases and increase mortality. The Gaussian diffusion model is easy to operate with faster calculation speed, lower basic data requirements, and high accuracy of long-term concentration simulation. Hence, this paper adopts the Gaussian diffusion model to obtain accurate long-term simulation quickly. Furthermore, there is little existing research on systematically accounting for PM2.5 concentration changes at airports caused by aircraft activities. The data in this study are from January 2016 to December 2023, covering more than 140 airports in China^50^ (the turnover accounts for more than 95% of the turnover of all airports in China), which can provide a scientific basis for environmental protection and health protection in China. The data in this study can be used to evaluate health impacts and emission policy. The research framework diagram of the methods part is shown in Fig. 1.

## Methods

### Collect detailed flight information and calculate PM2.5 emissions

This study collects monthly total turnover (domestic flights and foreign flights) data during the period of 2006 to 2023 from the Civil Aviation Administration of China website^51^, then calculates the proportion of turnover accounted for by each airport (average number of takeoffs and landings). Then finds the unit ton-kilometer consumption on the Civil Aviation Administration of China website^51^, the monthly fuel consumption of each airport is obtained. The PM2.5 emissions of each airport in the LTO stage of each month are calculated according to the modified BFFM2-FOA-FPM method^52^ and then averaged over time to the emission flow (that is, the number of days in the month is divided by 24 hours, then divided by 3600 seconds), the unit is g/s.

### Analyzing PM2.5 emission results

**Fig. 2:**
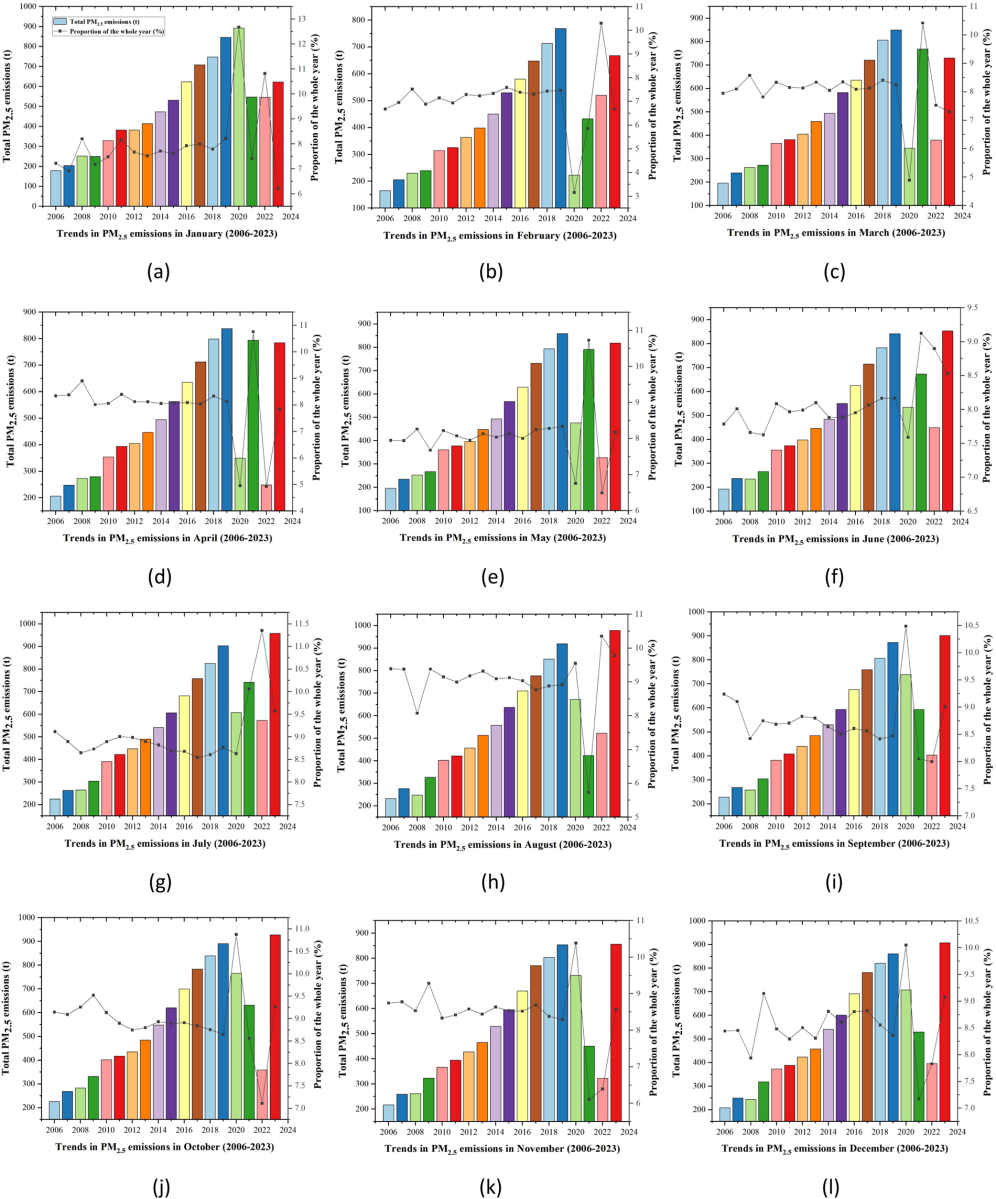
Trends in monthly PM2.5 emissions from January 2006 to December 2023. (**a**)Trends in PM2.5 emissions in January (2006–2023). (**b**) Trends in PM2.5 emissions in February (2006–2023). (**c**) Trends in PM2.5 emissions in March (2006–2023). (**d**) Trends in PM2.5 emissions in April (2006–2023). (**e**) Trends in PM2.5 emissions in May (2006–2023). (**f**) Trends in PM2.5 emissions in June (2006–2023). (**g**) Trends in PM2.5 emissions in July (2006–2023). (**h**) Trends in PM2.5 emissions in August (2006–2023). (**i**) Trends in PM2.5 emissions in September (2006–2023). (**j**) Trends in PM2.5 emissions in October (2006–2023). (**k**) Trends in PM2.5 emissions in November (2006–2023). (**l**) Trends in PM2.5 emissions in December (2006–2023).

This study employs the Modified BFFM2-FOA-FPM method^48^ to assess the total PM2.5 emissions originating from aviation activities in China. Consequently, Fig. 2 illustrates the distribution of monthly PM2.5 emissions spanning from January 2006 to December 2023, along with the corresponding annual proportion results. When evaluating the comprehensive annual emission trend, PM2.5 emissions manifest three discernible stages. Between 2006 and 2008, in tandem with the burgeoning development of the aviation industry, PM2.5 emissions experienced a rapid increase^53^. Subsequently, from the end of 2008 to 2019, the financial crisis induced economic downturns across various industries, including China’s aviation sector^54^. In the initial phase, the aviation industry’s sluggish growth contributed to a reduction in PM2.5 emissions^55^. In the later period, as the economy gradually recovered, the aviation industry underwent substantial expansion, leading to heightened fuel combustion and consequently increased PM2.5 emissions^56^.

From the end of 2019 to 2023, the large-scale spread of the new coronavirus significantly restricted people’s air travel, plunging China’s aviation industry into a trough and inflicting severe setbacks. The industry’s scale contracted sharply, resulting in a substantial reduction in PM2.5 emissions during this period— notably, PM2.5 emissions in 2020 amounted to only 7,042.64 tons, reflecting a 31.65% reduction compared to 2019 emissions. However, with the gradual improvement of the epidemic and the corresponding recovery of people’s demand for air travel, PM2.5 emissions from aviation resumed a rapid annual growth trend. Notably, PM2.5 emissions generated by China’s aviation in 2021 increased by 4.77% compared to 2020.

Additionally, when examining the 12-month average change trend, it becomes evident that aviation-related PM2.5 emissions in China are consistently higher in the second half of the year compared to the first half. Specifically, in Fig. 2, it is evident that August consistently exhibits the highest annual PM2.5 emissions, representing the most substantial proportion within the year. In contrast, PM2.5 emissions reach their lowest levels annually in February, constituting the minimum proportion. In August 2019, aviation-induced PM2.5 emissions peaked at 918.78 tons, comprising 8.92% of the annual emissions. Conversely, in February 2019, China’s aviation-related PM2.5 emissions were a mere 768.97 tons, constituting only 7.46% of the annual emissions. This substantial variance can be attributed to two primary factors. Primarily, August marks the peak season for tourism and air travel, potentially resulting in a significant surge in the operational capacity of the aviation industry. The heightened combustion of aviation fuel during this period releases increased PM2.5 particulate matter. Additionally, environmental conditions characterized by elevated temperatures and humidity hinder the dispersion of particulate matter, promoting the accumulation of emissions in the air and ultimately leading to the formation of PM2.5. Consequently, due to the synergistic impact of heightened aviation activities and unfavorable atmospheric conditions, Chinese aviation consistently contributes more to PM2.5 emissions in August compared to February.

Because this study measures PM2.5 emissions during the LTO stage, it has nothing to do with the flight distance but is directly related to the aircraft model and frequency of takeoffs and landings. One significant determinant of the variations in annual PM2.5 emissions is the diversity of aircraft types deployed. From 2006 to 2023, China’s aviation sector progressively diminished its reliance on regional aircraft in favor of wide-body and narrow-body aircraft characterized by higher emission intensity^57^. This transition is predominantly motivated by the sustained evolution of China’s aviation industry, propelled by the escalating demand for domestic medium and long-distance travel. Consequently, there is a notable shift towards the increased utilization of wide-body and narrow-body aircraft endowed with augmented passenger capacity and extended range capabilities. Notably, the most positive observation pertains to the comprehensive emission intensity of PM2.5 in 2018, which recorded the lowest numerical value at a mere 0.2772 g/kg. During this period, Chinese airlines predominantly utilized narrow-body aircraft with a moderate emission intensity, effectively managing PM2.5 emissions while catering to the ever-expanding demand for passenger travel.

### Calculate PM2.5 concentration by employing the Gaussian diffusion model

Then, the Gaussian diffusion model^58^ is used to calculate the average PM2.5 concentration of each airport in each month.

The detailed equations of Gaussian diffusion model are

1 $$C=\frac{E}{2\pi \mu {\delta }_{y}{\delta }_{z}}\times exp\left(\frac{-{y}^{2}}{2{\delta }_{y}^{2}}\right)\times P$$

2 $$P=2\,exp\left(\frac{-{h}^{2}}{2{\delta }_{z}^{2}}\right)+exp{\sum }_{n=1}^{2}\left[\frac{-{\left(nl-h\right)}^{2}}{2{\delta }_{z}^{2}}\right]+exp{\sum }_{n=1}^{2}\left[\frac{-{\left(nl+h\right)}^{2}}{2{\delta }_{z}^{2}}\right]$$

*C* is the PM2.5 concentration (μg/m^3^), *E* is the PM2.5 emission flow (g/s), *μ* is the wind speed (m/s), this study set *s μ* = 3.7. *δ*_*y*_, *δ*_*z*_ are the diffusion coefficient of the horizontal and vertical directions, this study sets *δ*_*y*_ = 1.33, *δ*_*z*_ = 1. *h* is the effective height. *n* is the number of smoke reflections, in this study, *h* = 2 and *n* = 3. *l* is the mixing layer height (m). Generally, for the coastal airports, *l* = 900, and for the other ones, *l*=1100. For the possible emission height that may have effects on humans *y*, this study selects *y* = 1, *y* = 2, and *y* = 3, and then calculate the average value as the final concentration.

### Analyzing PM2.5 concentration results

From 2006 to 2023, the PM2.5 concentration produced by China’s aviation sector demonstrated a clear overall upward trend (Supplementary Table 2). Despite the substantial impediment caused by the epidemic outbreak at the end of 2019, resulting in a steep decline in PM2.5 concentration data, subsequent efforts to control the epidemic led to a gradual recovery of PM2.5 concentration data. Throughout the entire period from 2006 to 2023, among the recorded highest PM2.5 concentration data at all airports, the zenith was observed in 2018 at 5.7985 μg/m^3^, while the nadir was registered in 2022 at 2.0574 μg/m^3^. This discovery suggests that the epidemic has, to a certain extent, mitigated pollution emissions within the aviation industry. However, it is imperative to acknowledge that this deceleration effect is likely temporary, and the aviation industry may gradually return to its usual operational levels post-epidemic, potentially resulting in a resurgence of emission levels.

Moreover, upon delving deeper into the temporal dynamics of PM2.5 concentration across individual airports monthly, this research discerns a prevailing trend. During the interval spanning January to August annually, the PM2.5 concentration near each airport consistently exhibits an ascending pattern, culminating in a zenith during August or September, succeeded by a gradual descent. This finding aligns cohesively with the PM2.5 emission patterns observed at each airport.

**Fig. 3:**
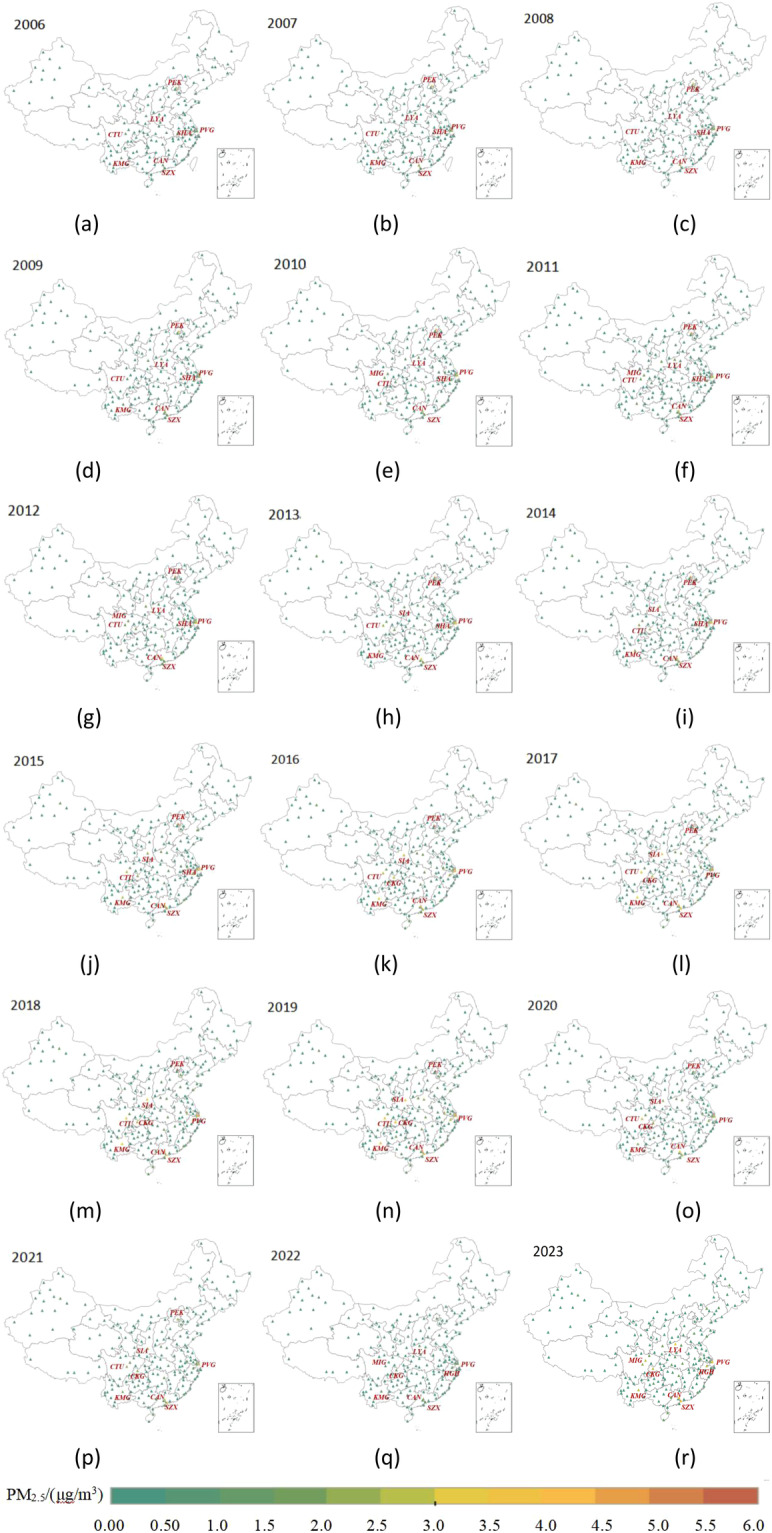
Annual average PM2.5 concentration caused by aviation activities at each airport from January 2006 to December 2023. (**a**) 2006. (**b**) 2007. (**c**) 2008. (**d**) 2009. (**e**) 2010. (**f**) 2011. (**g**) 2012. (**h**) 2013. (**i**) 2014. (**j**) 2015. (**k**) 2016. (**l**) 2017. (**m**) 2018. (**n**) 2019. (**o**) 2020. (**p**) 2021. (**q**) 2022. (**r**) 2023.

From 2006 to 2023, Fig. 3 delineates the eight airports exhibiting the highest annual average concentrations of PM2.5. These airports include Beijing Capital Airport (PEK), Shanghai Pudong Airport (PVG), Shanghai Hongqiao Airport (SHA), Guangzhou Baiyun Airport (CAN), Shenzhen Baoan Airport (SZX), Chengdu Shuangliu Airport (CYU), Kunming Changshui Airport (KMG), Xi’an Xianyang Airport (SIA), and Chongqing Jiangbei Airport (CKG). As shown in Fig. 3 (m), in the year 2018, the annual average PM2.5 concentrations at PEK, PVG, CYU, KMG, CAN, SZX, SIA, and CKG were recorded as 5.7985 μg/m^3^, 4.7670 μg/m^3^, 4.5079 μg/m^3^, 3.4070 μg/m^3^, 3.3610 μg/m^3^, 3.3253 μg/m^3^, 3.1208 μg/m^3^, and 2.8401 μg/m^3^, respectively. These airports are in densely populated and economically vibrant regions such as Beijing, Shanghai, Guangzhou, Chengdu, Shenzhen, Kunming, Xi’an, and Chongqing. Consequently, they experience heightened passenger and cargo demands, resulting in increased route activities and elevated PM2.5 concentrations originating from airport emissions.

### Policy recommendations for government departments and airlines

From the above conclusions, it is not difficult to find that, first, from the perspective of years, in the years after 2006, the PM2.5 concentration recorded at all airports generally showed an upward trend. This trend indicates a continued strong growth trajectory for the aviation industry. Therefore, government departments should introduce relevant environmental control policies to guide airlines to adopt a more green and sustainable development model, thereby effectively restraining the growth rate of PM2.5. Secondly, from the perspective of month, August and September are the months with the highest PM2.5 concentration. This is mainly related to the increase in air passengers traveling during the summer vacation. In response to this phenomenon, government departments should actively mobilize citizens to use more green and low-carbon travel methods to travel out, and airlines should also sell more special air tickets during the off-season to encourage citizens to travel during off-peak periods. Finally, from a regional perspective, densely populated areas with high economic development such as Beijing, Shanghai and Guangzhou have higher PM2.5 concentrations. This reminds government departments to set different emission reduction standards for airlines with reference to the economic development situation of different regions.

## Data Records

Our computed results have been meticulously documented in two files. The first, “Supplementary Table 1-Monthly PM2.5 flow.xlsx,” meticulously records the PM2.5 flow induced by aircraft at Chinese airports during the specified timeframe. The first column is the year and the second column of this document enumerates the corresponding three-character codes for each Chinese airport, as defined by the International Air Transport Association (IATA). The subsequent columns, spanning from the second to the thirteenth, encapsulate each airport’s monthly PM2.5 flow data. Similarly, the second file, titled “Supplementary Table 2-Monthly PM2.5 concentration.xlsx,” is a comprehensive summary of PM2.5 concentration values resulting from aircraft activities at Chinese airports within the mentioned timeframe. Mirroring the structure of the first document, the primary column designates the three-character codes for each airport, while the ensuing columns (second to thirteenth) meticulously outline the monthly PM2.5 concentration data produced by each airport.

## Technical Validation

### Accuracy analysis

In this section, we discuss the accuracy of the results. We did not find direct data on the concentration of PM2.5 emitted by airport aviation activities. We can only make comparisons through data from the China Environmental Inspection Station^59^. This article takes Guangzhou Baiyun Airport (CAN) as an example. There is a total of 12 monitoring stations within 50 km of Guangzhou Baiyun Airport, namely 1351 A, 1352 A, 1353 A, 1354 A, 1355 A, 1356 A, 1357 A, 1358 A, 1359 A, 1360 A, 1376 A, 1377 A, 1378 A, 1719A, 1720A, and 1721A. The distances from the airport are 28.61 km, 32.11 km, 33.45 km, 34.19 km, 49.33 km, 9.68 km, 29.05 km, 27.20 km, 26.45 km, 16.71 km, 46.45 km, 43.28 km, 41.92 km, 43.10 km, 46.19 km, and 36.46 km. This study uses the inverse distance method to weight the average monitoring data of each monitoring station (the farther the distance, the smaller the weight), and obtains the average PM2.5 concentration within 50 km of Baiyun Airport. This article takes June 2020 as an example. The average detection data at the testing station is 2.767 μg/m^3^. The calculated data in this article is 2.603 μg/m^3^, with an error rate of 5.9%. Considering that there may be other emission sources near the airport, the calculation results in this article have relatively high accuracy.

Furthermore, subject to missing data, this study has not considered the impacts of wind speed and seasons in different airports. Future research should set varying parameters to conduct more precise calculations.

### Comparisons with existing emission databases

There is little existing research on systematically accounting for PM2.5 concentration changes at airports caused by aircraft activities, so the results of this article are an excellent supplement to the current data.

### Supplementary information

Supplementary Table 1

Supplementary Table 2

## Data Availability

The data calculation is mainly done by Matlab 2014, and the code can be found in Supplementary Information.
